# Diabetes in Cushing’s disease: age, hypercortisolism, central adiposity, and β-cell stress as determinants of glycaemia

**DOI:** 10.3389/fendo.2026.1941836

**Published:** 2026-09-30

**Authors:** Przemysław Witek, Jakub Kwiatkowski, Joanna Witek, Katarzyna Szamotulska, Grzegorz Zieliński

**Affiliations:** 1 Department of Internal Medicine, Endocrinology and Diabetes, Medical University of Warsaw, Warsaw, Poland; 2 Department of Diabetology, Institute of Mother and Child, Warsaw, Poland; 3 Department of Epidemiology and Biostatistics, Institute of Mother and Child, Warsaw, Poland; 4 Department of Neurosurgery, Military Institute of Medicine, National Research Institute, Warsaw, Poland

**Keywords:** BMI, body composition, Cushing’s disease, diabetes, HbA1c, insulin resistance, metabolism

## Abstract

**Objective:**

Diabetes is a frequent complication of Cushing’s disease, but metabolic differences between normal glucose tolerance, prediabetes, and diabetes remain incompletely characterised. We aimed to characterise glucose metabolism across these groups, assess abnormalities in patients with normal glucose tolerance, and identify clinical, hormonal, and body-composition factors associated with glycaemia.

**Design:**

Cross-sectional analysis of a prospective cohort with matched healthy controls.

**Material and methods:**

The study included 50 patients with Cushing’s disease and 52 age- and sex-matched healthy controls who underwent detailed metabolic phenotyping, including multi-timepoint oral glucose tolerance testing with assessment of insulin sensitivity and β-cell compensatory function, and body composition analysis by dual-energy X-ray absorptiometry. Forward stepwise linear regression was performed separately in each group to identify variables independently associated with HbA1c.

**Results:**

Glucose metabolism disturbances affected 68% of patients with Cushing’s disease, including prediabetes in 42% and diabetes in 26%, with most diabetes cases previously undiagnosed. Even patients with normal glucose tolerance showed lower insulin sensitivity and enhanced β-cell secretory activity compared with healthy controls. Prediabetes was characterised mainly by lower insulin sensitivity with preserved β-cell compensation, whereas diabetes was associated with a reduced insulin-to-glucose AUC ratio without significantly lower insulin sensitivity than in prediabetes. In Cushing’s disease, age, morning cortisol, waist-to-hip ratio, and proinsulin were independently associated with HbA1c, explaining 59.5% of its variance; in controls, only age and BMI were associated with HbA1c. BMI was not significantly correlated with HbA1c in Cushing’s disease, whereas trunk fat percentage was positively associated with HbA1c.

**Conclusions:**

In Cushing’s disease, reduced insulin sensitivity with preserved β-cell compensation is detectable even in patients with normal glucose tolerance, whereas overt diabetes is characterised by impaired β-cell compensation without significantly lower insulin sensitivity than in prediabetes. Age, morning cortisol, waist-to-hip ratio, and proinsulin are independently associated with HbA1c. Central fat distribution may be more closely related to glycaemia than BMI.

## 1 Introduction

Cushing’s disease is a state of hypercortisolism caused by excessive secretion of adrenocorticotropic hormone (ACTH) by a pituitary adenoma and represents the most common cause of endogenous hypercortisolism (1). It accounts for approximately 70% of all cases of Cushing’s syndrome and occurs 3–8 times more frequently in women than in men, with a peak incidence in the third and fourth decades of life (2, 3). If left untreated, Cushing’s disease is associated with significantly increased mortality compared to the general population (4). Excess ACTH secretion leads to increased cortisol production by the adrenal cortex, resulting in a wide spectrum of systemic complications, including arterial hypertension, osteoporosis, thromboembolic events, alterations in body fat distribution, and glucose metabolism disturbances (5–8). Among these, abnormalities in glucose homeostasis represent one of the most frequent and clinically relevant metabolic consequences (9–11).

Hypercortisolism contributes to dysglycaemia through multiple mechanisms (9, 10, 12). Cortisol stimulates hepatic gluconeogenesis and promotes peripheral insulin resistance, particularly in skeletal muscle, which undergoes catabolic changes (10). At the molecular level, cortisol impairs insulin signalling by inhibiting key components of the insulin receptor pathway and glucose transporter type 4 (GLUT4) translocation (13, 14). Additionally, cortisol exerts direct effects on pancreatic β-cells, contributing to their dysfunction (15). As a result, impaired glucose tolerance is observed in up to 70% of patients, while overt diabetes mellitus develops in 20–45% of cases (5, 10).

Because diabetes in Cushing’s disease develops secondary to chronic hypercortisolism, it may present with some characteristic clinical and metabolic features and a particular pattern of progression (10, 11, 16). These include preferential visceral fat accumulation and predominantly postprandial glucose abnormalities, which may occur despite relatively preserved fasting glycaemia (17). Available studies further suggest that insulin resistance may already be present at the stage of normal glucose tolerance and initially compensated by increased insulin secretion, whereas progression towards prediabetes and overt diabetes may reflect deterioration of β-cell compensatory capacity rather than further worsening of insulin sensitivity (18, 19).

Despite these observations, metabolic and clinical differences between patients with normal glucose tolerance, prediabetes, and overt diabetes remain incompletely characterised in Cushing’s disease (10, 18, 19). Comparison of patients with Cushing’s disease who retain normal glucose tolerance with metabolically healthy individuals may additionally help identify early metabolic alterations associated with chronic hypercortisolism before clinically apparent dysglycaemia develops (18). In particular, the relative contribution of insulin resistance and β-cell dysfunction across these groups has not been fully established, while detailed metabolic phenotyping incorporating dynamic glucose testing and additional markers of β-cell function, such as C-peptide and proinsulin, remains limited (18). The relationships between cortisol excess, central adiposity, and glycaemic status also require further characterisation (9, 10). An additional limitation of existing studies is the heterogeneity of analysed cohorts, which frequently include mixed populations of patients with different forms of Cushing’s syndrome, including pituitary and adrenal causes, while studies focusing exclusively on Cushing’s disease remain relatively scarce (20, 21).

Therefore, this study aimed to characterise glucose metabolism across normal glucose tolerance, prediabetes, and diabetes in patients with Cushing’s disease, assess metabolic alterations in patients who retain normal glucose tolerance, and identify clinical, hormonal, and body-composition factors associated with glycaemia.

## 2 Materials and methods

### 2.1 Study design and participant selection

This cross-sectional analysis used baseline data from a prospective observational cohort of 50 consecutive adult patients diagnosed with Cushing’s disease according to the diagnostic criteria detailed below. All patients underwent transsphenoidal surgery and hospitalisation at the Department of Endocrinology of the Military Institute of Medicine in Warsaw. Patients were referred from endocrinology centres across Poland. All metabolic, hormonal, anthropometric, and body composition evaluations detailed below were performed during the same standardised preoperative assessment prior to transsphenoidal surgery. The control group comprised 52 age- and sex-matched healthy volunteers without clinical features raising suspicion of hypercortisolism on medical history and physical examination, including easy bruising, facial plethora, proximal muscle weakness, wide reddish-purple striae, or unexplained osteoporosis. The group was selected as a metabolically healthy reference population to assess metabolic abnormalities in patients with Cushing’s disease and normal glucose tolerance. General exclusion criteria for both groups included age <18 years, pregnancy or breastfeeding, active infection or severe acute illness, malignancy, severe renal or hepatic disease, uncontrolled thyroid disease, and use of medications known to interfere with hypothalamic–pituitary–adrenal axis function. Patients with Cushing’s disease were additionally excluded in cases of recurrent disease or non-pituitary hypercortisolism, whereas control participants were required to have no known disturbances of glucose metabolism and to demonstrate normal glucose tolerance at study assessment; two control participants with newly detected prediabetes were excluded.

All participants provided written informed consent. The study was approved by the Bioethics Committee of the Military Institute of Medicine in Warsaw (approval no. 54/WIM/13).

### 2.2 Anthropometric and body composition assessment

Body mass index (BMI) was calculated from weight and height measured after an overnight fast. Overweight was defined as BMI 25.0–29.9 kg/m^2^ and obesity as BMI ≥30.0 kg/m^2^. Waist and hip circumferences were measured at standard anatomical landmarks and waist-to-hip ratio (WHR) was calculated. Body composition was assessed using dual-energy X-ray absorptiometry (DXA; Hologic Delphi W, Hologic Inc., MA, USA) by the same technician. DXA-derived parameters included total fat mass and trunk fat mass, assessed both as absolute mass and as a percentage of the corresponding total or regional mass. The trunk region was defined by a horizontal upper boundary below the chin, lateral boundaries adjacent to the ribs, and oblique lower boundaries passing through the hip joints.

### 2.3 Glucose metabolism assessment

A detailed family history of type 2 diabetes in first-degree relatives was obtained. Information was recorded only when the participant was certain of the diagnosis. All available medical records were reviewed for previously diagnosed diabetes or prediabetes and current antidiabetic treatment. In all patients, fasting glucose, insulin, proinsulin, C-peptide, and HbA1c were measured. In patients without a prior diagnosis of diabetes, a 75-g oral glucose tolerance test (OGTT) was additionally performed with glucose and insulin measurements at 0, 30, 60, 90, and 120 minutes. Metformin was discontinued 7 days prior to testing. Patients with a previously established diagnosis of diabetes were classified in the diabetes group on the basis of documented medical history and did not undergo OGTT. Consequently, OGTT-derived variables were not available for these patients, who were therefore not included in analyses involving these variables.

Diabetes and prediabetes were classified according to the American Diabetes Association (ADA) criteria (22). Diabetes was defined as fasting plasma glucose ≥126 mg/dL (7.0 mmol/L), 2-hour plasma glucose ≥200 mg/dL (11.1 mmol/L) during a 75-g OGTT, or HbA1c ≥6.5%. Prediabetes was defined as fasting plasma glucose 100–125 mg/dL (5.6–6.9 mmol/L), 2-hour plasma glucose 140–199 mg/dL (7.8–11.0 mmol/L) during OGTT, or HbA1c 5.7–6.4%.

Insulin resistance was assessed indirectly using homeostasis model assessment of insulin resistance (HOMA-IR), quantitative insulin sensitivity check index (QUICKI), and the Matsuda index.

1. HOMA-IR was calculated as: fasting glucose (mmol/L) × fasting insulin (µU/mL)/22.5
2. QUICKI: 1/[log(fasting insulin) + log(fasting glucose)]
3. Matsuda index: 10,000/√[(fasting glucose × fasting insulin) × (mean glucose × mean insulin during OGTT)]

Area under the curve (AUC) values were calculated using the trapezoidal method. The insulin-to-glucose AUC ratio (AUC I/G) was used as a surrogate marker of stimulated β-cell secretory function (23, 24).

### 2.4 Hormonal and neuroradiological assessment

The diagnosis of endogenous Cushing’s syndrome was based on standard biochemical testing. First-line assessment included 24-hour urinary free cortisol (UFC), the 1-mg overnight dexamethasone suppression test (DST), and late-night salivary cortisol (LNSC). Abnormal findings included elevated 24-hour UFC (>75 µg/24 h), failure of serum cortisol to suppress to <1.8 µg/dL following the 1-mg overnight DST, or elevated LNSC (>0.27 µg/dL) in at least two separate measurements.

Cases were considered diagnostically equivocal when first-line biochemical test results were discordant, with different tests providing conflicting evidence for hypercortisolism despite persistent clinical suspicion of Cushing’s syndrome. In such cases, late-night serum cortisol was assessed as a second-line test, either during sleep or after quiet awakening. Serum cortisol concentrations <1.8 µg/dL during sleep or <7.5 µg/dL after awakening were considered to exclude Cushing’s syndrome.

ACTH-dependent hypercortisolism was confirmed by unsuppressed plasma ACTH >20 pg/mL. Differentiation between pituitary and ectopic ACTH secretion was based on high-dose DST (≥50% suppression of urinary free cortisol [UFC] or urinary 17-hydroxycorticosteroids [17-OHCS]) and corticotropin-releasing hormone (CRH) stimulation testing (intravenous CRH 100 µg or 1 µg/kg body weight; an ACTH increase >50% and a cortisol increase >20% from baseline within 60 minutes were considered consistent with Cushing’s disease). Pituitary origin was confirmed by magnetic resonance imaging (MRI). Patients with completely normal pituitary imaging were excluded. Inferior petrosal sinus sampling (IPSS) was performed in cases with inconclusive or discordant findings.

Only UFC, the 1-mg overnight DST, and LNSC were used for the initial biochemical assessment of hypercortisolism. Late-night serum cortisol was used as a second-line test in diagnostically equivocal cases, whereas plasma ACTH, high-dose DST, CRH stimulation testing, pituitary MRI, and IPSS were used subsequently to establish ACTH dependence and determine the source of ACTH excess. A summary of the biochemical and diagnostic tests used in the assessment of hypercortisolism and determination of the pituitary origin of ACTH excess is provided in **Supplementary Table S1**.

For the purposes of the present study, hormonal parameters included morning (08:00) serum cortisol, midnight serum cortisol, cortisol after the 1-mg overnight DST, plasma ACTH and 24-hour UFC.

### 2.5 Laboratory measurements

All hormonal and metabolic parameters were measured using standardised laboratory methods. Plasma ACTH was measured by two-step immunoradiometric assay (IRMA; ELSA-ACTH, CIS Bio International, France; sensitivity 2 pg/mL). Serum cortisol was measured by electrochemiluminescence immunoassay (ECLIA; Elecsys 2010, Roche Diagnostics, Switzerland; sensitivity 0.02 µg/dL). 24-hour urinary free cortisol (UFC) was measured by radioimmunoassay following dichloromethane extraction (RIA; Cortisol RIA kit, Immunotech, Czech Republic; sensitivity 1.8 µg/24h). Plasma glucose was measured using the enzymatic hexokinase method (Cobas c501, Roche Diagnostics, USA). Glycated haemoglobin (HbA1c) was determined by immunoturbidimetry (AU680, Beckman Coulter, USA). Serum insulin was measured by chemiluminescent immunoassay (CLIA; LIAISON Insulin, DiaSorin, Italy; sensitivity 0.23 µU/mL; no cross-reactivity with proinsulin). C-peptide was measured by chemiluminescent immunoassay (LIAISON C-peptide, DiaSorin, Italy; sensitivity 0.01 ng/mL). Proinsulin was determined by radioimmunoassay (RIA; EMD Millipore, USA; sensitivity 3.0 pmol/L).

### 2.6 Statistical analysis

Statistical analyses were performed using IBM SPSS Statistics version 23.0 and R software version 4.5.3. Descriptive statistics included mean, median, standard deviation, proportions, and correlation coefficients. Between-group comparisons were performed using Student’s t-test for normally distributed variables or the Mann–Whitney U test for non-normally distributed variables. For comparisons involving more than two groups, analysis of variance (ANOVA) or the Kruskal–Wallis test was applied, as appropriate. *Post-hoc* comparisons following ANOVA used Tukey HSD test; following Kruskal–Wallis, Dunn’s test with Holm correction was applied. Categorical variables were compared using Fisher’s exact test. Associations between continuous variables were assessed using Pearson’s correlation coefficient or Spearman’s rank correlation coefficient, as appropriate. In subgroup analyses, nonparametric exact tests were applied. The AUC was calculated using the trapezoidal method, and differences between AUC values were assessed using the Mann–Whitney U test.

To identify variables independently associated with HbA1c, forward stepwise linear regression was performed separately in patients with Cushing’s disease and in healthy controls. Candidate variables were age, sex, BMI, WHR, trunk fat percentage, morning serum cortisol, ACTH, C-peptide, proinsulin, hypertension, and family history of type 2 diabetes. These variables were selected *a priori* based on clinical relevance and biological plausibility. Variables were entered sequentially according to the Akaike Information Criterion (AIC). Model fit was assessed using adjusted R^2^, multicollinearity using variance inflation factors (VIF), and residual normality using the Shapiro–Wilk test. A *p*-value < 0.05 was considered statistically significant.

## 3 Results

### 3.1 Comparison of patients with Cushing’s disease and controls

Patients with Cushing’s disease and healthy controls were well-matched for age and sex (**Table 1**). Patients with Cushing’s disease had significantly higher BMI (30.75 ± 6.45 vs. 24.46 ± 3.38 kg/m^2^, *p* < 0.001), with a substantially higher prevalence of obesity (48.0% vs. 5.8%, *p* < 0.001), greater waist circumference, hip circumference, and WHR (all *p* < 0.001). Hypertension was more prevalent in the Cushing’s group (78.0% vs. 15.4%, *p* < 0.001). Abnormal glucose metabolism was present in 68.0% of patients (prediabetes 42.0%, diabetes 26.0%), with 10 of 13 diabetic patients newly diagnosed. Glucose-lowering therapy was used in 26 patients (52.0%), most commonly metformin (mean dose 1395 ± 753 mg/day). Family history of type 2 diabetes did not differ between groups (*p* = 0.826).

**Table 1:** Clinical and metabolic characteristics of patients with Cushing’s disease and controls.

Parameter	Cushing’s disease (*n* = 50)	Control group (*n* = 52)	*p-*value
Female sex, *n* (%)	39 (78.0%)	42 (80.8%)	0.73
Age, years (mean ± SD)	42.38 ± 14.86	44.10 ± 13.28	0.54
BMI, kg/m^2^ (mean ± SD)	30.75 ± 6.45	24.46 ± 3.38	<0.001
BMI category, *n* (%)			<0.001
Normal weight	8 (16.0%)	34 (65.4%)	
Overweight	18 (36.0%)	15 (28.8%)	
Obesity	24 (48.0%)	3 (5.8%)	
Waist circumference, cm (median [IQR])	105.50 (99.13–118.75)	88.00 (81.00–93.25)	<0.001
Hip circumference, cm (median [IQR])	103.00 (97.00–114.75)	93.00 (86.50–99.25)	<0.001
WHR (median [IQR])	1.01 (0.97–1.07)	0.95 (0.90–0.98)	<0.001
Hypertension, *n* (%)	39 (78.0%)	8 (15.4%)	<0.001
Glycaemic status (OGTT-based), *n* (%)			<0.001
Normal glucose tolerance	16 (32.0%)	52 (100.0%)	
Prediabetes	21 (42.0%)	0 (0.0%)	
Diabetes	13 (26.0%)	0 (0.0%)	
Glucose-lowering therapy, *n* (%)	26 (52%)	0 (0%)	<0.001
Metformin	20 (76.9%)		
Metformin + sulfonylurea	3 (11.5%)		
Metformin + insulin	3 (11.5%)		
Family history of type 2 diabetes, *n* (%)			0.826
None	33 (66.0%)	36 (69.2%)	
Mother	10 (20.0%)	10 (19.2%)	
Father	4 (8.0%)	5 (9.6%)	
Both parents	3 (6.0%)	1 (1.9%)	
Serum cortisol (8.00), µg/dL (median [IQR])	24.36 (18.62–28.25)	12.49 (10.66–17.89)	<0.001
ACTH (8.00), pg/mL (median [IQR])	67.15 (46.73–90.68)	19.65 (11.25–24.25)	<0.001
Total body mass, kg (mean ± SD)	83.49 ± 18.98	69.25 ± 12.73	<0.001
Total fat mass, kg (mean ± SD)	34.22 ± 10.36	22.28 ± 6.70	<0.001
Trunk mass, kg (mean ± SD)	40.69 ± 8.96	31.61 ± 6.39	<0.001
Trunk fat mass, kg (mean ± SD)	17.31 ± 5.07	9.60 ± 4.06	<0.001
Total fat mass, % (mean ± SD)	40.5 ± 5.2	32.1 ± 7.2	<0.001
Trunk fat mass, % (mean ± SD)	42.0 ± 5.6	29.8 ± 7.8	<0.001
Glucose (0 min), mg/dl (mean ± SD)	95.76 ± 17.29	87.07 ± 7.59	0.002
Glucose (120 min), mg/dl (mean ± SD)	154.98 ± 56.57^††^	90.53 ± 22.25	<0.001
Mean glucose, mg/dl (mean ± SD)	157.66 ± 42.80^††^	106.79 ± 21.26	<0.001
Glucose AUC (OGTT), mg·min/dL (mean ± SD)	19919.02 ± 5572.29^††^	13355.19 ± 2917.82	<0.001
HbA1c, % (mean ± SD)	5.91 ± 0.80	5.18 ± 0.28	<0.001
Insulin (0 min), µIU/mL (median [IQR])	13.60 (10.85–19.95)^†^	9.05 (5.98–11.05)	<0.001
Insulin (120 min), µIU/mL (median [IQR])	94.15 (48.65–174.075)^††^	30.25 (19.25–49.55)	<0.001
Mean insulin, µIU/mL (median [IQR])	79.27 (44.315–117.66)^††^	42.38 (32.53–53.31)	<0.001
Insulin AUC (OGTT), µIU·min/mL (median [IQR])	9756.75 (5957.63–14897.63)^††^	5758.50 (4207.50–7400.25)	<0.001
C-peptide, ng/mL (median [IQR])	4.50 (3.30–5.40)^†^	1.90 (1.70–2.30)	<0.001
Proinsulin, pmol/L (median [IQR])	26.10 (15.25–39.48)^†^	6.49 (3.53–9.30)	<0.001
HOMA-IR (median [IQR])	3.35 (2.42–4.55)^†^	1.94 (1.27–2.54)	<0.001
QUICKI (median [IQR])	0.32 (0.31–0.33)^†^	0.35 (0.33–0.37)	<0.001
Matsuda (median [IQR])	2.75 (1.66–3.58)^††^	5.43 (3.93–7.86)	<0.001

^†^ Data available for 47 patients; ^††^data available for 46 patients.

Data are presented as number (% of total), mean ± SD or median (IQR). Continuous variables were compared using the Student’s t-test or Mann–Whitney U test, as appropriate. Categorical variables were compared using the χ^2^ test or Fisher’s exact test, as appropriate.

ACTH–adrenocorticotropic hormone; AUC–area under the curve; BMI–body mass index; DXA–dual-energy X-ray absorptiometry; HbA1c–glycated haemoglobin; HOMA-IR–homeostasis model assessment of insulin resistance; IQR–interquartile range; *n*–number; OGTT–oral glucose tolerance test; QUICKI–quantitative insulin sensitivity check index; SD–standard deviation; WHR–waist-to-hip ratio.

Serum cortisol and ACTH levels were significantly higher in patients with Cushing’s disease (both *p <* 0.001). Sex-stratified analyses of hormonal parameters within the Cushing’s group did not reveal significant differences (**Supplementary Table S2**). DXA confirmed markedly altered body composition in the Cushing’s group across all measured parameters (all *p <* 0.001).

Compared with healthy controls, patients with Cushing’s disease had higher glucose and insulin concentrations during OGTT, higher glucose and insulin AUC, HbA1c, C-peptide, proinsulin, and HOMA-IR, together with lower QUICKI and Matsuda indices (all *p* ≤ 0.002).

### 3.2 Metabolic and clinical characteristics by glycaemic status

Patients were stratified by glycaemic status into normal glucose tolerance (NGT), prediabetes, and diabetes (**Table 2**). There were no significant differences in BMI, body fat distribution, or family history of type 2 diabetes. Age tended to be higher in the diabetes group (*p* = 0.057). Diabetes was exclusively observed in women (*p* = 0.036).

**Table 2:** Clinical and metabolic characteristics of patients with Cushing’s disease according to glycaemic status.

Parameter	Cushing NGT (*n* = 16)	Cushing prediabetes (*n* = 21)	Cushing diabetes (*n* = 13)	*p-*value (among Cushing subgroups)	Control group (*n* = 52)	*p-*value (Cushing NGT vs. control)
Age, years (mean ± SD)	39.81 ± 11.25	39.14 ± 16.81	50.77 ± 12.98	0.057	44.10 ± 13.28	0.212
Female sex, *n* (%)	10 (62.5%)	16 (76.2%)	13 (100%)	0.036	42 (80.8%)	0.178
BMI, kg/m^2^ (mean ± SD)	29.55 ± 6.96	31.63 ± 6.42	30.82 ± 6.14	0.632	24.46 ± 3.38	0.011
Family history of T2DM, *n* (%)	6 (37.5%)	7 (33.3%)	4 (30.8%)	1.000	16 (30.8%)	0.761
Total fat mass, % (mean ± SD)	38.88 ± 5.58	40.7 ± 5.65	42.29 ± 3.59	0.216	32.10 ± 7.19	0.001
Trunk fat mass, % (mean ± SD)	39.82 ± 6.33	42.28 ± 5.74	44.15 ± 3.39	0.110	29.80 ± 7.76	<0.001
Glucose (0 min), mg/dl (mean ± SD)	84.19 ± 6.48^a^	97.38 ± 15.86^b^	107.39 ± 20.47^b^	0.001	87.08 ± 7.59	0.146
Glucose (120 min), mg/dl (mean ± SD)	98.69 ± 21.70^a^	160.33 ± 17.68^b^	242.56 ± 35.99^†c^	<0.001	90.53 ± 22.25	0.203
Mean glucose, mg/dl (mean ± SD)	118.28 ± 26.07^a^	165.79 ± 26.48^b^	208.69 ± 32.70^†c^	<0.001	106.80 ± 21.26	0.123
Glucose AUC (OGTT), mg·min/dL (mean ± SD)	14998.13 ± 3667.97^a^	21002.86 ± 3756.27^b^	26138.33 ± 4373.74^†c^	<0.001	13355.19 ± 2917.82	0.116
HbA1c, % (mean ± SD)	5.34 ± 0.39^a^	5.90 ± 0.70^b^	6.57 ± 0.82^c^	<0.001	5.18 ± 0.28	0.131
Insulin (0 min), µIU/mL (median [IQR])	12.00 (11.20–17.18)	17.00 (11.10–22.10)	15.15 (10.10–20.23)^††^	0.420	9.05 (5.97–11.05)	0.001
Insulin (120 min), µIU/mL (median [IQR])	64.95 (23.50–93.88)^a^	176.00 (66.60–252.40)^b^	65.90 (53.30–135.00)^†ab^	0.003	30.25 (19.25–49.55)	0.072
Mean insulin, µIU/mL (median [IQR])	64.44 (49.52–86.47)	107.80 (60.14–169.70)	41.84 (34.96–81.14)^†^	0.051	42.38 (32.53–53.31)	0.004
Insulin AUC (OGTT), µIU·min/mL (median [IQR])	9081.75 (6898.13–11311.83)	13198.50 (7918.50–20715.00)	5155.50 (4350.00–9826.50)^†^	0.051	5758.50 (4207.50–7400.25)	0.004
AUC insulin/glucose (median [IQR])	0.59 (0.45–0.73)^a^	0.64 (0.31–1.11)^a^	0.26 (0.17–0.36)^†b^	0.013	0.45 (0.34–0.57)	0.017
C-peptide, ng/mL (median [IQR])	4.10 (2.83–4.73)	4.50 (3.40–5.40)	4.00 (3.33–5.68)^††^	0.501	1.90 (1.70–2.30)	<0.001
Proinsulin, pmol/L (median [IQR])	17.61 (14.23–27.67)	36.61 (17.33–44.20)	28.15 (16.53–42.64)^††^	0.179	6.48 (3.53–9.30)	<0.001
HOMA-IR (median [IQR])	2.69 (2.10–3.49)	3.81 (2.49–4.70)	3.92 (2.59–5.36)^††^	0.093	1.94 (1.27–2.54)	0.003
QUICKI (median [IQR])	0.33 (0.32–0.34)	0.31 (0.30–0.33)	0.31 (0.30–0.33)^††^	0.093	0.35 (0.33–0.37)	0.003
Matsuda (median [IQR])	3.46 (2.56–4.72)^a^	2.03 (1.48–3.00)^b^	3.28 (1.44–3.51)^†ab^	0.011	5.43 (3.93–7.86)	0.002

^†^ Data available for 9 patients; ^††^Data available for 10 patients.

Data are presented as number (% of total), mean ± SD or median (IQR). For between-group comparisons among Cushing's disease subgroups, continuous variables were compared using one-way ANOVA or the Kruskal–Wallis test, as appropriate; *post-hoc* comparisons following ANOVA used Tukey HSD test and following Kruskal–Wallis used Dunn's test with Holm correction. For comparisons between Cushing NGT and the control group, Student's t-test or Mann–Whitney U test was applied, as appropriate. Categorical variables were compared using Fisher's exact test. Different superscript letters (a, b, c) indicate statistically significant differences between subgroups based on *post-hoc* testing.

AUC–area under the curve; BMI–body mass index; HbA1c–glycated haemoglobin; HOMA-IR–homeostasis model assessment of insulin resistance; IQR–interquartile range; *n*–number; NGT–normal glucose tolerance; OGTT–oral glucose tolerance test; QUICKI–quantitative insulin sensitivity check index; SD–standard deviation; T2DM–type 2 diabetes mellitus.

Glucose-related parameters, including fasting glucose, 120-min glucose, mean glucose during OGTT, glucose AUC, and HbA1c, increased across subgroups, with significant differences in all measured parameters. Insulin at 120 minutes was numerically highest in the prediabetes group and significantly higher than in NGT (*p* = 0.003). Mean insulin and insulin AUC showed a similar pattern at borderline significance (both *p* = 0.051). The AUC I/G was significantly lower in diabetes than in both NGT and prediabetes (*p* = 0.013), and the Matsuda index was significantly lower in prediabetes than in NGT (*p* = 0.011). Fasting insulin, C-peptide, proinsulin, HOMA-IR, and QUICKI did not differ significantly between subgroups.

Despite comparable glucose parameters (fasting glucose, 120-min glucose, mean glucose during OGTT, glucose AUC, and HbA1c), Cushing NGT patients had significantly higher fasting and mean insulin levels, insulin AUC, C-peptide, proinsulin, HOMA-IR, and AUC I/G, whereas QUICKI and Matsuda indices were lower than in controls.

**Figure 1** presents the distribution of patients across glycaemic subgroups and healthy controls according to the Matsuda index and AUC I/G.

**Figure 1:**
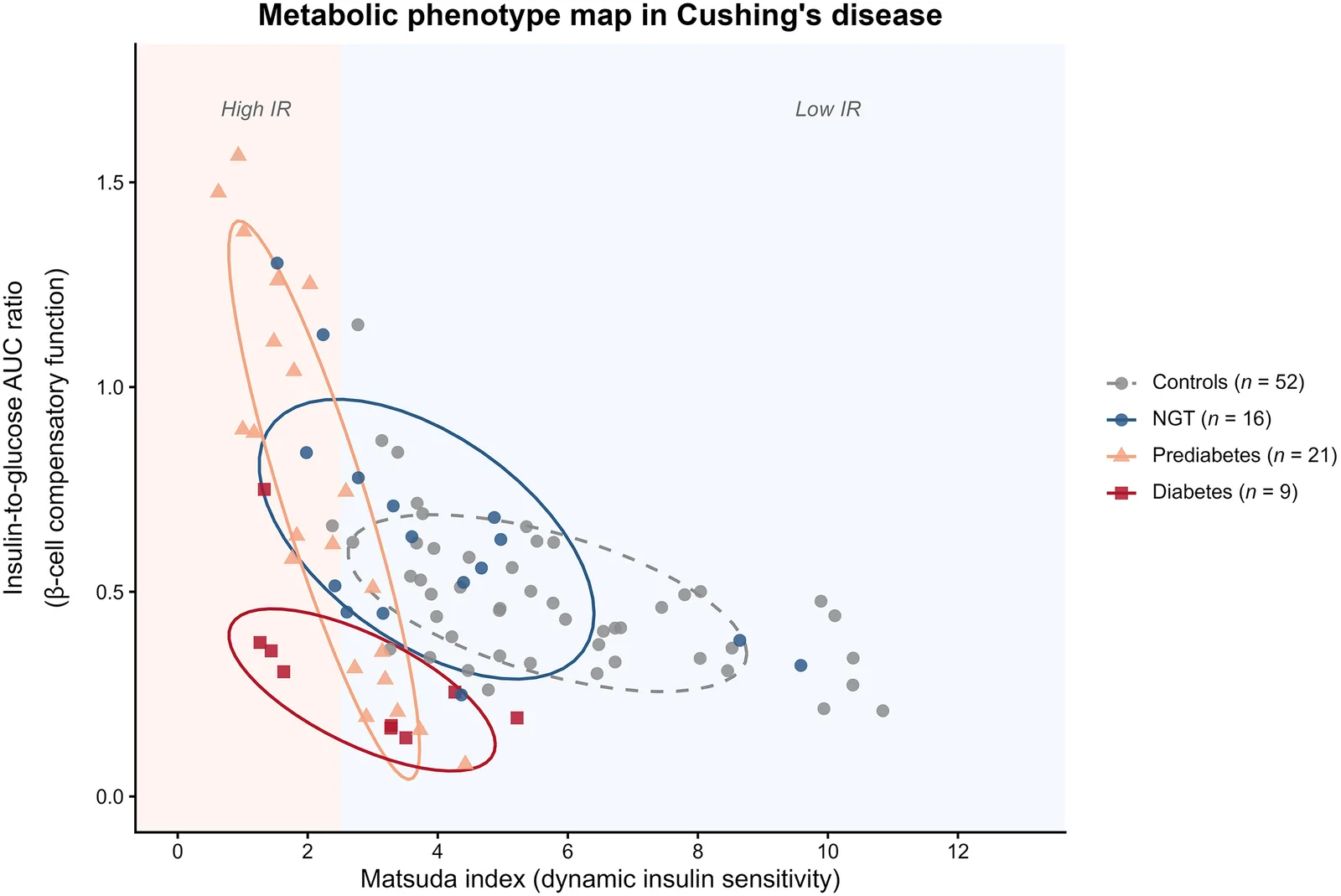
Metabolic phenotype map illustrating the distribution of Cushing's disease glycaemic subgroups and healthy controls according to dynamic insulin sensitivity (Matsuda index) and stimulated β-cell secretory function (insulin-to-glucose AUC ratio). Ellipses represent 68% confidence regions around the mean of each group based on a bivariate normal approximation. Background shading (orange/blue) delineates regions of higher and lower insulin resistance (Matsuda index cut-off of 2.5), respectively. AUC, area under the curve; IR, insulin resistance; NGT, normal glucose tolerance.

### 3.3 Metabolic parameters by BMI category

In patients with Cushing’s disease, fasting and OGTT-derived glucose concentrations were numerically highest in the overweight group, with fasting, mean glucose and glucose AUC significantly higher than in normal weight. In contrast, HbA1c and insulin-related parameters (fasting insulin, mean insulin, insulin AUC, C-peptide, proinsulin) increased progressively with BMI and were significantly higher in obesity than in normal weight (**Table 3**). Insulin sensitivity also worsened across categories, with significantly higher HOMA-IR and lower QUICKI and Matsuda index in obesity than in normal weight.

**Table 3:** Glucose metabolism parameters according to BMI categories in patients with Cushing’s disease and controls.

	Cushing’s disease				Control group			
Parameter	Normal weight (*n* = 8)	Overweight (*n* = 18)	Obesity (*n* = 24)	*p-*value	Normal weight (*n* = 34)	Overweight (n = 15)	Obesity (n = 3)	*p-*value
Glucose (0 min), mg/dl (mean ± SD)	83.38 ± 6.59^a^	101.28 ± 18.08^b^	95.75 ± 17.45^ab^	0.048	85.88 ± 7.55	89.00 ± 7.89	91.00 ± 4.36	0.277
Glucose (120 min), mg/dl (mean ± SD)	124.00 ± 47.28	176.71 ± 61.23^††^	149.19 ± 50.97^†^	0.074	87.00 ± 21.93	100.73 ± 21.49	79.66 ± 17.04	0.092
Mean glucose, mg/dl (mean ± SD)	127.93 ± 41.49^a^	176.48 ± 45.20^††b^	153.74 ± 34.84^†ab^	0.022	103.86 ± 20.41	112.71 ± 23.83	110.46 ± 15.68	0.395
Glucose AUC (OGTT), mg·min/dL (mean ± SD)	16078.13 ± 5569.79^a^	22332.35 ± 5903.66^††b^	19428.57 ± 4474.24^†ab^	0.024	12986.47 ± 2814.98	14060.00 ± 3260.45	14010.00 ± 2292.94	0.465
HbA1c, % (mean ± SD)	5.23 ± 0.46^a^	5.98 ± 0.8^ab^	6.09 ± 0.81^b^	0.026	5.07 ± 0.23^a^	5.34 ± 0.28^b^	5.57 ± 0.06^b^	<0.001
Insulin (0 min), µIU/mL (median [IQR])	10.30 (9.48–11.48)^a^	13.75 (10.73–17.08)^ab^	17.80 (12.40–23.70)^†b^	0.005	9.55 (5.58–11.15)	8.50 (6.45–10.90)	9.00 (7.65–9.95)	0.756
Insulin (120 min), µIU/mL (median [IQR])	44.95 (26.95–82.63)^a^	114.00 (53.30–151.60)^††ab^	96.60 (64.20–252.40)^†b^	0.049	26.40 (17.10–38.30)^a^	46.90 (33.70–66.75)^b^	31.20 (27.00–43.05)^ab^	0.036
Mean insulin, µIU/mL (median [IQR])	46.52 (30.88–57.87)^a^	80.52 (41.84–107.80)^††ab^	90.40 (64.52–156.18)^†b^	0.007	37.31 (28.66–49.53)	47.70 (39.91–59.46)	56.70 (52.75–67.87)	0.050
Insulin AUC (OGTT), µIU·min/mL (median [IQR])	6086.25 (3711.38–7853.25)^a^	9687.00 (5155.50–13198.50)^††ab^	11970.00 (9046.50–20289.00)^†b^	0.005	5109.00 (3854.63–7042.88)	6345.00 (5213.25–7977.75)	7587.00 (7152.00–9468.75)	0.092
C-peptide, ng/mL (median [IQR])	2.50 (2.33–3.43)^a^	4.40 (3.33–4.78)^ab^	4.70 (3.70–5.80)^†b^	0.008	1.85 (1.63–2.10)	2.10 (1.85–2.35)	2.30 (2.10–2.45)	0.048
Proinsulin, pmol/L (median [IQR])	6.30 (3.10–12.93)^a^	19.73 (15.72–38.18)^b^	37.61 (26.10–47.27)^†c^	<0.001	6.00 (3.38–7.88)	6.57 (3.35–8.40)	11.90 (11.45–19.95)	0.071
HOMA-IR (median [IQR])	2.08 (1.96–2.32)^a^	3.29 (2.53–4.09)^b^	3.83 (2.79–5.79)^†b^	0.002	2.02 (1.15–2.49)	1.89 (1.40–2.56)	1.96 (1.67–2.27)	0.635
QUICKI (median [IQR])	0.342 (0.336–0.345)^a^	0.320 (0.311–0.332)^b^	0.313 (0.297–0.328)^†b^	0.002	0.343 (0.333–0.375)	0.347 (0.332–0.363)	0.345 (0.338–0.354)	0.635
Matsuda (median [IQR])	4.53 (4.02–6.08)^a^	2.59 (1.98–3.38)^††b^	1.79 (1.33–3.15)^†b^	0.002	6.21 (4.04–9.43)	4.78 (3.87–6.25)	4.96 (4.17–5.05)	0.150

^†^ Data available for 21 patients; ^††^Data available for 17 patients.

Data are presented as mean ± SD or median (IQR). Continuous variables were compared using one-way ANOVA or the Kruskal–Wallis test, as appropriate. *Post-hoc* comparisons following ANOVA used Tukey HSD test; following Kruskal–Wallis, Dunn's test with Holm correction was applied. Different superscript letters (a, b, c) indicate statistically significant differences between subgroups based on *post-hoc* testing.

AUC, area under the curve; HbA1c, glycated haemoglobin; HOMA-IR, homeostasis model assessment of insulin resistance; IQR, interquartile range; *n*, number; OGTT, oral glucose tolerance test; QUICKI, quantitative insulin sensitivity check index; SD, standard deviation.

In the control group, the only parameters differing significantly across BMI categories were HbA1c, insulin at 120 minutes, and C-peptide.

### 3.4 Correlation analyses

Correlation analyses revealed markedly different patterns between groups (**Figure 2**). In the control group, anthropometric and body composition parameters (BMI, total fat percentage, trunk fat percentage, and waist circumference) showed consistent moderate-to-strong positive correlations with glucose-related variables (fasting glucose, glucose AUC, and HbA1c), particularly HbA1c, whereas in Cushing’s disease these associations were weak and largely non-significant, with only trunk fat percentage showing a modest correlation with HbA1c. Conversely, in Cushing’s disease, all anthropometric and body composition parameters showed strong positive correlations with insulin-related variables and HOMA-IR and strong inverse correlations with the Matsuda index. In controls, similar associations were present but weaker. No significant correlations were observed between morning cortisol or ACTH and glucose- or insulin-related parameters in Cushing’s disease.

**Figure 2:**
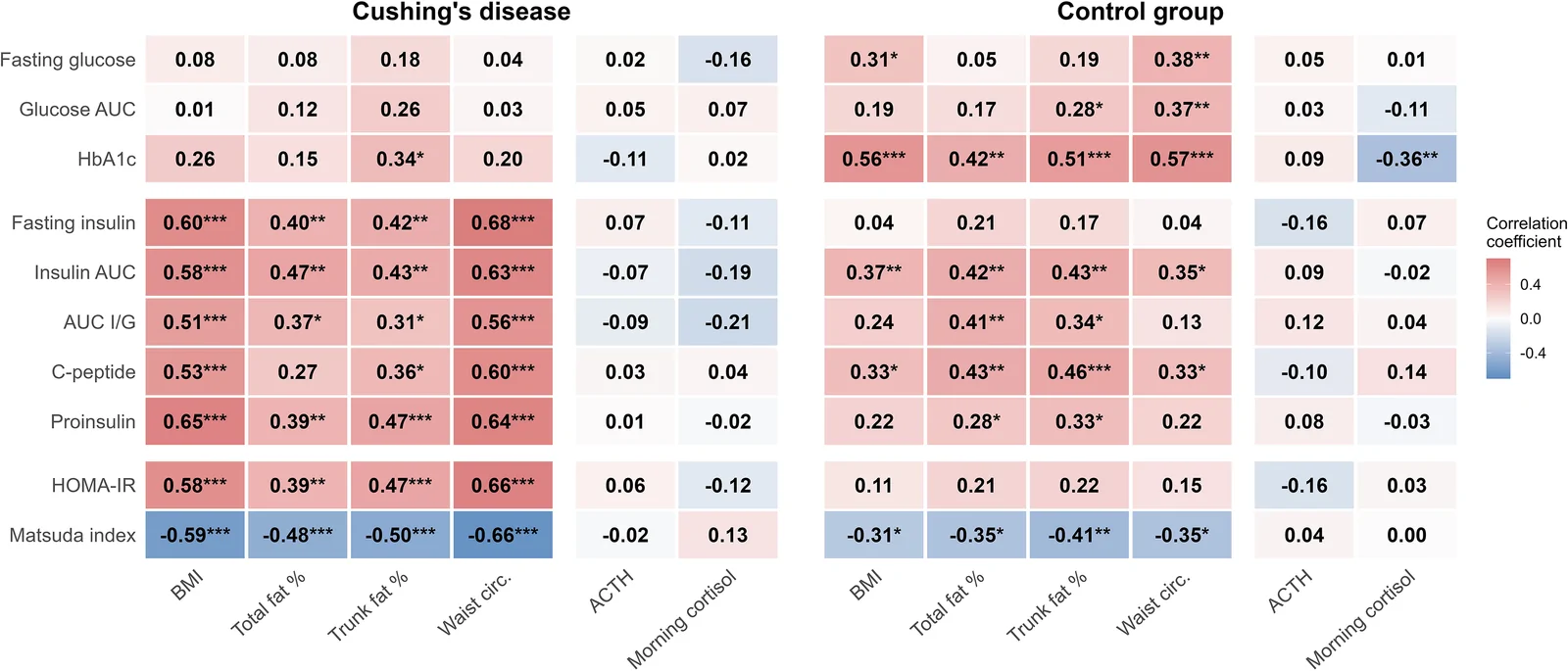
Heatmaps showing correlations between anthropometric/body composition parameters, HPA axis hormones, and glucose- or insulin-related metabolic parameters in patients with Cushing’s disease and healthy controls. Pearson correlation coefficients are shown for associations between body composition parameters and glucose-related variables (fasting glucose, glucose AUC, and HbA1c); Spearman correlation coefficients are presented for all remaining associations, including insulin-related parameters, insulin resistance indices, and HPA axis hormones (cortisol, ACTH). Asterisks indicate statistical significance (*p < 0.05, **p < 0.01, ***p < 0.001). ACTH, adrenocorticotropic hormone; AUC I/G, insulin-to-glucose area under the curve ratio; BMI, body mass index; HbA1c, glycated haemoglobin; HOMA-IR, homeostatic model assessment of insulin resistance; HPA, hypothalamic–pituitary–adrenal.

### 3.5 Factors associated with HbA1c in patients with Cushing’s disease

To identify variables independently associated with HbA1c in patients with Cushing’s disease, forward stepwise linear regression was performed. Age, morning serum cortisol, WHR, and proinsulin were included in the final model, which explained 59.5% of the variance in HbA1c (adj. R^2^ = 0.595, *p* < 0.001; **Table 4**). All four variables were statistically significant in the final model, with VIF values <1.25. BMI, trunk fat percentage, ACTH, C-peptide, sex, hypertension, and family history of type 2 diabetes were evaluated as candidate variables but were not included in the final model.

**Table 4:** Forward stepwise linear regression analysis predicting HbA1c in patients with Cushing's disease (*n* = 47).

Variable	B	95% CI	β	*p*-value
Age	0.028	[0.019, 0.037]	0.589	<0.001
Morning cortisol	0.026	[0.012, 0.039]	0.382	<0.001
WHR	3.453	[1.323, 5.582]	0.340	0.002
Proinsulin	0.010	[0.002, 0.017]	0.263	0.012

Adj. R^2^ = 0.595, *p* < 0.001. Candidate variables selected *a priori* from the following pool: age, BMI, WHR, trunk fat percentage, morning serum cortisol, ACTH, proinsulin, C-peptide, sex, hypertension, and family history of type 2 diabetes. All VIF values < 1.25, indicating no multicollinearity. Model assumptions confirmed by Shapiro–Wilk test (W = 0.984, *p* = 0.780).

B, unstandardised regression coefficient; CI, confidence interval; β, standardised regression coefficient; WHR, waist-to-hip ratio; VIF, variance inflation factor.

Morning serum cortisol (rho = +0.02, *p* = 0.887) and WHR (r = +0.22, *p* = 0.134) were not significantly associated with HbA1c when examined individually in univariable analyses. However, both variables were significantly associated with HbA1c after adjustment for the other variables included in the final regression model.

Prior metformin use was assessed as a potential confounder in a sensitivity analysis. Its inclusion did not alter the significance of the four variables identified in the primary model (adj. R^2^ = 0.613), while metformin itself was not significantly associated with HbA1c (*p* = 0.095; **Supplementary Table S3**).

In healthy controls, forward stepwise linear regression using the same set of candidate variables identified age and BMI as the only variables independently associated with HbA1c (adj. R^2^ = 0.422, *p* < 0.001; **Supplementary Table S4**). Morning serum cortisol, WHR, and proinsulin were not included in the final control model, unlike in patients with Cushing’s disease (**Figure 3**).

**Figure 3:**
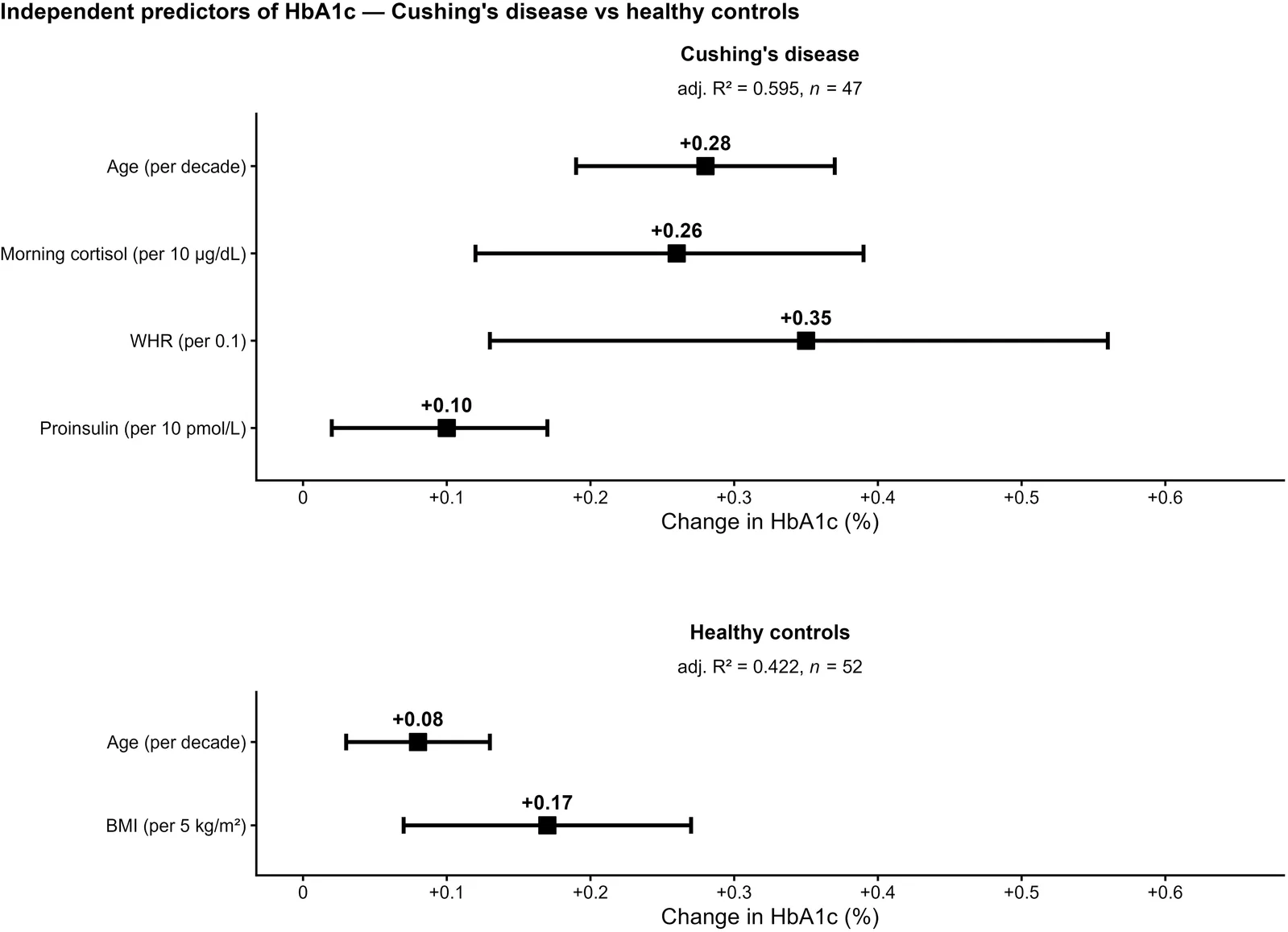
Regression coefficients (B) with 95% confidence intervals for independent predictors of HbA1c in patients with Cushing's disease (*n* = 47; upper panel) and healthy controls (*n* = 52; lower panel), scaled to clinically meaningful units: age per decade, morning cortisol per 10 µg/dL, WHR per 0.1 unit, proinsulin per 10 pmol/L, and BMI per 5 kg/m^2^. Predictors were selected independently for each group by forward stepwise linear regression. BMI, body mass index; CI, confidence interval; HbA1c, glycated haemoglobin; WHR, waist-to-hip ratio.

## 4 Discussion

Our findings indicate that metabolic alterations are present even in patients with Cushing’s disease who retain normal glucose tolerance. In this subgroup, reduced insulin sensitivity was observed alongside preserved β-cell compensation. Overt diabetes, compared with prediabetes, was characterised by impaired β-cell compensation without significantly lower insulin sensitivity. In addition, age, morning cortisol, WHR, and proinsulin were independently associated with HbA1c, suggesting that glycaemic status in Cushing’s disease may be shaped by the combined influence of hormonal exposure, central adiposity, and β-cell stress.

Glucose metabolism disturbances were highly prevalent in Cushing’s disease, with 42.0% of patients presenting with prediabetes and 26.0% with diabetes, the majority previously undiagnosed, underscoring the importance of systematic OGTT-based screening during diagnostic workup (10, 11). Baseline characteristics were consistent with previous reports, including a predominantly female cohort (78%) and markedly elevated BMI compared to controls (18, 19, 21, 25, 26).

Despite the high prevalence of dysglycaemia, no association was observed between family history of type 2 diabetes and glycaemic status, suggesting that this conventional risk marker may have limited discriminatory value in Cushing’s disease (27). This contrasts with Giordano et al. (19), although direct comparison is limited given the retrospective design and absence of detailed family history methodology in that study.

Age, however, appeared to remain an important correlate of glycaemic status. Older age showed a borderline association with diabetes (*p* = 0.057), consistent with both the general population (28) and previous studies in Cushing’s syndrome (19). A higher prevalence of diabetes among women was observed, contrasting with a meta-analysis of patients with Cushing’s disease that showed no significant sex differences (29). Nevertheless, unlike age, sex was not independently associated with HbA1c in the multivariable model, suggesting that this observation more likely reflects the demographic characteristics of our cohort than a true sex-specific association with glycaemic status.

To further characterise the metabolic phenotype across normal glucose tolerance, prediabetes, and diabetes, we examined insulin sensitivity and β-cell compensatory function (**Figure 1**). Both insulin resistance and enhanced β-cell secretory activity were already detectable in patients with normal glucose tolerance compared with healthy controls, indicating that metabolic abnormalities related to hypercortisolism are present even in patients who retain normal glucose tolerance (18). Among patients with Cushing’s disease, prediabetes was characterised mainly by lower insulin sensitivity with preserved β-cell compensation, whereas diabetes was associated with a reduced insulin-to-glucose AUC ratio without significantly lower insulin sensitivity than in prediabetes. These findings suggest that impaired β-cell compensation is an important feature of overt diabetes in Cushing’s disease, consistent with previous studies (18, 19, 30).

Among the evaluated insulin resistance indices, only the Matsuda index, which integrates both fasting and post-load insulin sensitivity, differed significantly between glycaemic subgroups, whereas fasting-based measures, including HOMA-IR and QUICKI, did not. This suggests that the Matsuda index may be more informative for detecting differences in insulin sensitivity in Cushing’s disease, consistent with the predominantly postprandial impairment of glucose metabolism associated with glucocorticoid excess (18). The considerable interindividual variability in the insulin-to-glucose AUC ratio within the prediabetes group suggests substantial differences in β-cell compensatory capacity between patients and warrants further investigation (31).

BMI did not differ across glycaemic subgroups, consistent with prior reports (18, 25). This finding is notable because, in type 2 diabetes, higher BMI has been associated with progressively poorer glycaemic control (32). In our cohort, however, there was no clear increase in fasting or OGTT-derived glucose concentrations with increasing BMI, and glucose values were numerically highest in overweight rather than obese patients. This may partly reflect the limited ability of BMI to capture the characteristic body-composition changes of hypercortisolism, in which central fat accumulation coexists with catabolic effects on skeletal muscle and bone (33–35). Interestingly, BMI correlated positively with insulin-related parameters, including the insulin-to-glucose AUC ratio, whereas this ratio was lower in patients with overt diabetes. This pattern raises the hypothesis that better preserved β-cell compensation in some patients with higher BMI may contribute to maintaining glycaemic control despite insulin resistance, potentially explaining the absence of a clear increase in fasting and OGTT-derived glucose concentrations with increasing BMI in our cohort (9).

Although morning serum cortisol was not significantly correlated with HbA1c in the univariable analysis, it was independently associated with HbA1c in the multivariable model. This suggests that the relationship between cortisol excess and glycaemia may depend on the broader metabolic context, including central adiposity and β-cell secretory stress. This interpretation is biologically plausible, as cortisol exerts diabetogenic effects through multiple mechanisms (13–15), whereas compensatory hyperinsulinaemia may buffer its net glycaemic effect. The absence of direct correlations between ACTH, UFC, or cortisol and metabolic parameters is consistent with prior reports (18, 19) and further suggests that circulating cortisol levels alone may not fully determine metabolic severity, which is likely modulated by additional factors including duration of exposure and local cortisol metabolism via 11β-hydroxysteroid dehydrogenase type 1 (11β-HSD1) (11, 19, 36–40). A recent study using continuous glucose monitoring (CGM) in autonomous cortisol secretion reported associations between cortisol burden and glycaemic variability, suggesting that CGM may detect alterations not captured by OGTT (41).

Forward stepwise regression identified age, morning cortisol, WHR, and proinsulin as variables independently associated with HbA1c in Cushing’s disease, jointly explaining 59.5% of its variance. Notably, neither cortisol nor WHR showed significant simple correlations with HbA1c, but both were independently associated with HbA1c in the multivariable analysis, suggesting that their relationships with glycaemia are better captured when other metabolic factors are considered simultaneously. In healthy controls, only age and BMI were associated with HbA1c, whereas in Cushing’s disease WHR, rather than BMI, showed an independent association with HbA1c, suggesting that central adiposity may be more informative than BMI in relation to glycaemia in the setting of chronic hypercortisolism.

This interpretation is supported by complementary analyses of anthropometric and body composition measures. BMI showed a strong positive correlation with HbA1c in controls (r = +0.557, *p* < 0.001), consistent with data from the general population including morbidly obese individuals (42, 43), but no such association was observed in Cushing’s disease (r = +0.26, *p* = 0.171). In contrast, trunk fat percentage, which can be assessed by DXA to characterise body fat distribution (44), correlated with HbA1c in patients with Cushing’s disease. This finding suggests that fat distribution may be more relevant to glycaemia than overall adiposity in Cushing’s disease.

Proinsulin, a biomarker of early β-cell dysfunction reflecting cellular stress and impaired proinsulin-to-insulin conversion, was also independently associated with HbA1c. Higher proinsulin levels may therefore indicate less efficient β-cell compensation, in which increased secretory demand is accompanied by impaired insulin processing and higher glycaemia (45, 46). Age was the only factor associated with HbA1c in both Cushing’s disease and controls, although the association was stronger in Cushing’s disease (+0.28% vs. +0.08% HbA1c per decade), suggesting that the glycaemic effects of ageing may be amplified by chronic hypercortisolism.

The main strengths of this study include prospectively collected data, a homogeneous cohort restricted to Cushing’s disease, detailed metabolic phenotyping with multi-timepoint OGTT and DXA-based body composition assessment, standardised laboratory methods, and an age- and sex-matched control group with full metabolic evaluation, enabling formal comparison of factors associated with HbA1c between groups. Limitations include the small sample size, inherent to the rarity of the condition, single-centre recruitment, and the cross-sectional nature of the analysis, which precludes causal inference and does not allow temporal changes across glycaemic categories to be established. Disease duration could not be reliably established and was therefore not included in analyses. Biochemical screening with the 1-mg DST was not performed in the control group; therefore, asymptomatic or oligosymptomatic hypercortisolism cannot be excluded with absolute certainty. In addition, the exclusion of control participants with screening-detected prediabetes resulted in a metabolically healthier comparator group than the general population and may have increased the apparent between-group differences. Therefore, the findings should be interpreted as comparisons between patients with Cushing’s disease and metabolically healthy individuals, not as evidence of differences between diabetes in Cushing’s disease and conventional type 2 diabetes.

## 5 Conclusions

In Cushing’s disease, reduced insulin sensitivity with preserved β-cell compensation is detectable even in patients with normal glucose tolerance. Overt diabetes is characterised by impaired β-cell compensation without significantly lower insulin sensitivity than that observed in prediabetes. Age, morning cortisol, WHR, and proinsulin are independently associated with HbA1c in Cushing’s disease, while central fat distribution may better reflect glycaemic abnormalities than BMI. These findings support longitudinal studies to determine how glucose dysregulation in Cushing’s disease evolves over time and after treatment of hypercortisolism.

## Data Availability

The raw data supporting the conclusions of this article will be made available by the authors, without undue reservation.
