# Supplementary material for: Diabetes in Cushing’s disease: age, hypercortisolism, central adiposity, and β-cell stress as determinants of glycaemia
Source: Front Endocrinol (Lausanne). 2026 Sep 30;17:1941836. doi: 10.3389/fendo.2026.1941836 (PMC13662288; doi:10.3389/fendo.2026.1941836)
Supplement: Supplementary file 1 [file Table1.docx]

**Table S1.** Summary of biochemical and diagnostic tests used in the assessment of hypercortisolism and determination of the pituitary origin of ACTH excess.

| **Test or assessment** | **Role in the diagnostic pathway** | **Interpretation used in the study** |
| --- | --- | --- |
|  |  |  |
| 24-hour urinary free cortisol (UFC) | First-line assessment of hypercortisolism | UFC >75 µg/24 h was considered elevated |
| 1-mg overnight dexamethasone suppression test (DST) | First-line assessment of hypercortisolism | Serum cortisol ≥1.8 µg/dL was considered failure to suppress |
| Late-night salivary cortisol (LNSC) | First-line assessment of hypercortisolism | LNSC >0.27 µg/dL in at least two separate measurements was considered elevated |
| Late-night serum cortisol during sleep | Second-line assessment in diagnostically equivocal cases | Serum cortisol <1.8 µg/dL was considered to exclude Cushing’s syndrome |
| Late-night serum cortisol after quiet awakening | Second-line assessment in diagnostically equivocal cases | Serum cortisol <7.5 µg/dL was considered to exclude Cushing’s syndrome |
| Plasma ACTH | Assessment of ACTH dependence | Unsuppressed ACTH >20 pg/mL was considered consistent with ACTH-dependent hypercortisolism |
| High-dose DST | Differentiation between pituitary and ectopic ACTH secretion | ≥50% suppression of UFC or urinary 17-hydroxycorticosteroids (17-OHCS) was used in the differentiation of pituitary and ectopic ACTH secretion |
| CRH stimulation test | Differentiation between pituitary and ectopic ACTH secretion | ACTH increase >50% and cortisol increase >20% from baseline within 60 min were considered consistent with Cushing’s disease |
| Pituitary MRI | Identification of a pituitary source | Used to confirm pituitary origin; patients with completely normal pituitary imaging were excluded |
| Inferior petrosal sinus sampling (IPSS) | Further source localisation | Performed in cases with inconclusive or discordant findings |

ACTH–adrenocorticotropic hormone; CRH–corticotropin-releasing hormone; DST–dexamethasone suppression test; IPSS–inferior petrosal sinus sampling; LNSC–late-night salivary cortisol; MRI–magnetic resonance imaging; UFC–urinary free cortisol; 17-OHCS–17-hydroxycorticosteroids.

**Table S2.** HPA axis hormonal parameters in patients with Cushing’s disease.

| **Parameter** | **Cushing’s disease (*n* = 50)** | ***p*-value** |
| --- | --- | --- |
|  |  |  |
| Cortisol (8.00, serum), µg/dL (median [IQR])  Female  Male | 24.36 (18.62–28.25)  25.00 (19.95–28.10)  19.25 (17.50–28.29) | 0.261 |
| ACTH (8.00), pg/mL (median [IQR])  Female  Male | 67.15 (46.73–90.68)  67.40 (50.30–96.40)  66.90 (43.15–86.15) | 0.412 |
| Cortisol (0.00, serum), µg/dL (median [IQR])  Female  Male | 16.00 (13.20–19.25)^†^  15.90 (13.16–18.63)  17.59 (13.92–20.64) | 0.774 |
| Cortisol (8.00, 1-mg overnight dexamethasone suppression test), µg/dL (median [IQR])  Female  Male | 14.22 (6.88–22.23)  16.30 (7.05–22.45)  13.00 (6.80–21.45) | 0.770 |
| Urinary free cortisol (24-hour urine collection), µg/24h (median [IQR])  Female  Male | 267.40 (210.50–426.85)  263.00 (205.40–390.15)  351.00 (213.00–459.00) | 0.504 |

^†^Data available for 49 patients

Data are presented as median (interquartile range, IQR). Between-group comparisons were performed using the Mann–Whitney U test.

ACTH–adrenocorticotropic hormone; HPA–hypothalamic-pituitary-adrenal; IQR–interquartile range; *n*–number

**Table S3.** Sensitivity analysis of the primary regression model additionally adjusted for prior metformin use in patients with Cushing's disease (*n* = 47).

| **Variable** | **B** | **95% CI** | **β** | ***p*-value** |
| --- | --- | --- | --- | --- |
| Age | 0.024 | [0.014, 0.034] | 0.505 | <0.001 |
| Morning cortisol | 0.022 | [0.008, 0.036] | 0.330 | 0.002 |
| WHR | 2.837 | [0.630, 5.044] | 0.279 | 0.013 |
| Proinsulin | 0.009 | [0.001, 0.016] | 0.241 | 0.020 |
| Prior metformin use | 0.272 | [−0.049, 0.593] | 0.192 | 0.095 |

Adj. R² = 0.613, *p* < 0.001. The primary regression model (age, morning serum cortisol, WHR, and proinsulin) was refitted with additional adjustment for prior metformin use to assess its potential confounding effect.

B–unstandardized regression coefficient; 95% CI–95% confidence interval for B; β–standardized regression coefficient; WHR–waist-to-hip ratio

**Table S4.** Forward stepwise linear regression analysis predicting HbA1c in healthy controls (*n* = 52).

| **Variable** | **B** | **95% CI** | **β** | ***p*-value** |
| --- | --- | --- | --- | --- |
| Age | 0.008 | [0.003, 0.013] | 0.395 | 0.001 |
| BMI | 0.034 | [0.015, 0.053] | 0.409 | <0.001 |

Adj. R² = 0.422, *p* < 0.001. Candidate variables selected a priori from the following pool (the same as in Cushing’s disease): age, BMI, WHR, trunk fat percentage, morning serum cortisol, ACTH, proinsulin, C-peptide, sex, hypertension, and family history of type 2 diabetes. All VIF values < 1.20, indicating no multicollinearity. Model assumptions confirmed by Shapiro–Wilk test (W = 0.970, *p* = 0.215).

B–unstandardized regression coefficient; BMI–body mass index; CI–confidence interval; β–standardized regression coefficient; VIF–variance inflation factor
